# Hypoadrenocorticism in cats: a 40-year update

**DOI:** 10.1177/1098612X241248381

**Published:** 2024-09-26

**Authors:** Magdalena J Glebocka, Alisdair Boag

**Affiliations:** The Royal (Dick) School of Veterinary Studies and The Roslin Institute, The University of Edinburgh, Easter Bush Veterinary Campus, Midlothian, Roslin, UK

**Keywords:** Hypoadrenocorticism, Addison’s disease, feline hypoadrenocorticism, primary hypoadrenocorticism

## Abstract

**Practical relevance:**

Addison’s disease is a very rare condition in cats, with only approximately 40 cases documented in the past 40 years since it was first described in 1983.

**Clinical challenges:**

While canine hypoadrenocorticism is a well-recognised disorder with clear diagnostic and treatment guidelines, feline hypoadrenocorticism remains a challenge because of its rarity and waxing and waning clinical signs. Furthermore, empirical treatment with corticosteroids, resulting in clinical improvement, contributes to delays in achieving the diagnosis and initiating treatment. Feline hypoadrenocorticism is diagnosed with an adrenocorticotropic hormone (ACTH) stimulation test; a low resting cortisol concentration with an inadequate or absent response to synthetic ACTH is diagnostic. Various ACTH stimulation-testing protocols are reported in published cases, with the majority using three time-limited blood samples. This can be limiting clinically, depending on cats’ clinical presentation and behaviour at the veterinary practice and tolerance for procedures. Long-term treatment, similar to canine hypoadrenocorticism, consists of oral corticosteroids, with several formulations licensed in the UK, and mineralocorticoids (desoxycorticosterone pivalate), of which the only available formulation (Zycortal; Dechra) is licensed for dogs and its safety has not been assessed in cats.

**Global importance:**

Feline hypoadrenocorticism occurs worldwide. Although no breed, sex or age association has been reported, cats aged <6 years are overrepresented.

## Introduction

Hypoadrenocorticism (also known as Addison’s disease) is a relatively rare, but well recognised condition in people and dogs. Addison’s disease is a very rare condition in cats, with only 48 cases documented in the literature since it was initially described in 1983.^1–29^

## Aetiopathogenesis

Primary hypoadrenocorticism is a consequence of adrenal cortex dysfunction. The most common cause of this has unknown pathophysiology; however, an autoimmune process is considered most likely, resulting in the insufficient production of either glucocorticoids, mineralocorticoids or both.^2^ Lymphocytic infiltration of the adrenal cortex on post-mortem histological examination has been reported in two cats, supporting an autoimmune-mediated process in cats.^1,3,4,29^ Adrenal cortex destruction and atrophy secondary to primary and metastatic lymphoma has been reported in two cats.^10^ Two patients with trauma-induced hypoadrenocorticism have been described.^6,8^ Adrenal insufficiency has also been reported in two kittens aged <12 months and congenital disease was hypothesised.^21,25^ Acute adrenal necrosis associated with neutrophilic and macrophagic inflammation was recently reported in a young cat.^28^

**Figure 1:**
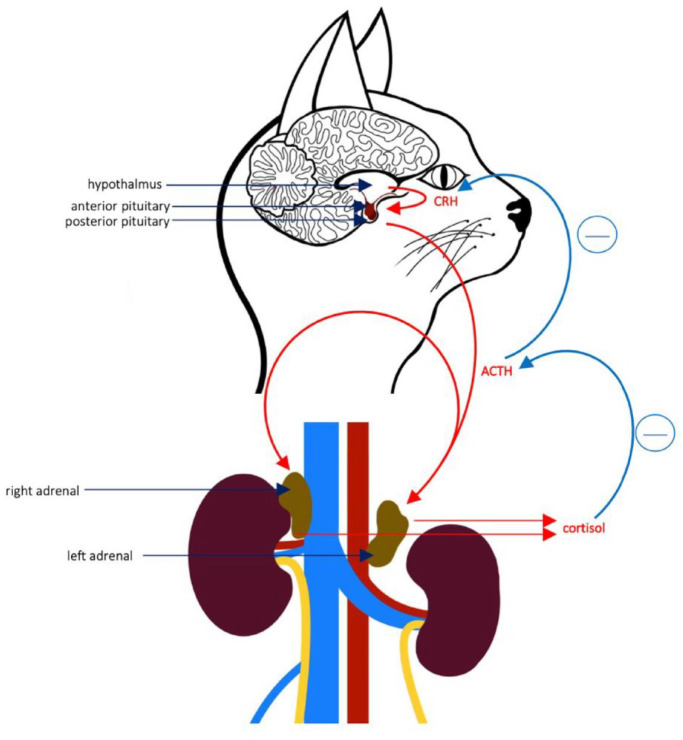
Pituitary adrenal axis in a cat. ACTH = adrenocorticotropic hormone; CRH = corticotropin releasing hormone

Secondary hypoadrenocorticism results from an inadequate secretion of adrenocorticotropic hormone (ACTH) from the pituitary gland (Figure 1). This is commonly reported to be caused by iatrogenic suppression of ACTH secretion through administration of corticosteroids and progesterone or sudden termination of a long-term treatment with steroids (steroid withdraw). Cats treated with methylprednisolone acetate (20 mg/week, SC for 1–4 weeks) had low to low-normal resting cortisol serum concentrations with an inadequate response to ACTH stimulation.^30^ Similar observations were noted in cats that were administered oral prednisolone at a dosage of 2 mg/kg q24h or higher for at least 14 days. In the latter cats, serum cortisol concentrations normalised within 30 days after termination of glucocorticoid therapy.^31^ Transient severe adrenocortical suppression has also been reported secondary to administration of the synthetic progestogen megestrol acetate.^31,32^ Furthermore, a single case of a T-cell-rich lymphocytic panhypophysitis resulting in bilateral adrenal cortex atrophy in a cat was reported in 2015.^19^

## Signalment

Signalment information was not available for all cats. The age of cats reported with Addison’s disease ranged from seven months to 14 years (median 4 years) (n = 38). Neutered female (n = 14/38) and neutered male (n = 20/38) cats were similarly affected; four of the cats were entire females (n = 4/38). Entire males were not reported. The majority of the cats were reported as either domestic shorthair or longhair cats (n = 26/39), followed by British shorthair cats (n = 9/39), one Birman (n = 1/39), one Siamese (n = 1/39), one crossbreed cat (n = 1/39) and one Bengal cat (n = 1/39).

## Clinical signs

Commonly reported clinical signs and physical examination findings, collated from reports of feline hypoadrenocorticism published between 1983 and 2023, are listed in Tables 1 and 2, respectively.^1–29^ The onset of clinical signs varies from days to months; clinical signs tend to wax and wane, and are usually milder in patients with a longer clinical course.

**Table 1:** Most common clinical signs collated from reports of feline hypoadrenocorticism published between 1983 and 2023^1–29^

Clinical signs		Number of cases
Gastrointestinal	Anorexia	25/48
	Vomiting	14/48
	Weight loss	14/48
	Constipation	9/48
	Adipsia	2/48
	Polyphagia	1/48
	Hyporexia	2/48
Neurological	Weakness	9/48
	Ataxia	3/48
	Dysphagia	2/48
	Empty swallowing	2/48
	Hindlimb weakness	1/48
	Difficulty jumping/climbing	1/48
	Clinginess	1/48
	Bilateral mydriasis	1/48
Other	Lethargy	25/48
	Polyuria/polydipsia	13/48
	Hypothermia	3/48
	Previous response to steroid therapy	2/48
	Waxing and waning clinical signs	2/48
	Hair loss	1/48
	Periuria	1/48

**Table 2:** Most common physical examination findings collated from reports of feline hypoadrenocorticism published between 1983 and 2023^1–29^

Physical examination findings	Number of cases
Dehydration	24/48
Hypothermia	11/48
Weak pulse	8/48
Slow capillary refill time	5/48
Lateral recumbency	4/48
Collapse	3/48
Heart murmur	3/48
Bradycardia	2/48
Painful abdomen	2/48
Tachypnoea	1/48
Seizures	1/48

Acute onset of hypoadrenocorticism consistent with adrenal crisis carries a guarded prognosis. Cats usually present collapsed, with severe dehydration and marked muscle weakness.

## Diagnostics

### Laboratory findings

Routine laboratory findings in most reported cases were abnormalities expected with mineralocorticoid deficiency, including hypochloraemia, hyponatraemia and hyperkalaemia (Table 3).^1–29^ Azotaemia (30/48) and hyperphosphataemia (11/48) are likely a result of volume depletion and decreased renal perfusion. A decreased sodium to potassium (Na:K) ratio (<27) was described in 12/48 of the reported cases and was normal in 10/48 cases (Table 4). In a study evaluating a decreased Na:K ratio in 49 cats, hypoadrenocorticism was not diagnosed in any cases.^24^ Anaemia and neutrophilia were the most commonly seen haematological abnormalities with 11/48 and 7/48 reported cases, respectively. Leukocytosis and lack of stress leukogram are unusual in a sick cat and should raise a suspicion of adrenal insufficiency; catecholamine release resulting in an increased lymphocyte count is the primary differential explanation. Urine specific gravity (USG) was assessed in 24 of the reported cases, with a USG <1.030 in 14 samples.^1–29^ The cause of an inadequate renal concentrating ability is poorly understood; however, it has been hypothesised to be secondary to renal sodium loss resulting in a medullary washout.^33^

**Table 3:** Most common blood work abnormalities collated from reports of feline hypoadrenocorticism published between 1983 and 2023^1–29^

Blood work abnormalities		Number of cases
Haematology	Anaemia	11/48
	Neutrophilia	7/48
	Lymphocytosis	6/48
	Lack of stress leukogram	4/48
Biochemistry	Azotaemia	30/48
	Elevated creatinine kinase	9/48
	Increased ALT	6/48
Electrolytes	Hyponatraemia	32/48
	Hyperkalaemia	27/48
	Hypochloraemia	14/48
	Hyperphosphataemia	11/48
	Na:K ratio normal	10/48
	Na:K ratio <27*	12/48

* Historically, a Na:K ratio <27 has been used clinically as indicative for hypoadrenocorticism in canine medicine. It was first used in feline medicine in the 1980s when the first cases were diagnosed and has been used since in reviews, case reports and studies^3,20,21,24,27,34^

ALT = alanine aminotransferase; Na:K = sodium:potassium

**Table 4:** Median values and ranges for sodium, potassium, chloride and Na:K ratio^1–28^

Electrolytes	Median value (range)
Sodium (Na^2+^) (mmol/l)	132 (113–156)
Potassium (K^+^) (mmol/l)	5.8 (3.6–8)
Chloride (Cl^–^) (mmol/l)	100 (90.8–124)
Na:K ratio	23.3 (15.12–27.3)

Na:K = sodium:potassium

### Imaging

Radiographic findings were consistent with lung hypoperfusion and microcardia (n = 15/18).^1,4,8,11,15,20,25^ Thoracic imaging was reportedly normal in only 3/18 cats with confirmed hypoadrenocorticism.^12,24^ Adrenal glands with a measurement below the reference interval (RI) on ultrasound examination were reported in 9/18 cats that underwent an abdominal ultrasonographic examination.^17,24,26,28,29^ Left adrenal gland measurements were in the range of 1.2–3.9 mm (median 2.85 mm) and those of the right adrenal gland were in the range of 1.8–5.1 mm (median 2.7 mm). In one case, the right adrenal gland could not be visualised.^17^ The RI for adrenal gland measurements in cats varies based on modality. Ultrasonographic measurements show average length of 10.4 mm and 10.8 mm with average height of 3.6 mm and 3.7 mm (left and right adrenal gland respectively).^35^ When assessed using CT the mean (± SD) length was 11.6 (± 2.1) mm and height was 6.1 (± 1.3) mm.^36^ Bilateral marked adenomegaly with ultrasonographic measurements up to 1 cm in thickness was reported in one cat.^28^

### Endocrine testing

Endocrine testing should be performed to confirm a suspected initial diagnosis of hypoadrenocorticism in cats. Hypoadrenocorticism is diagnosed with an ACTH stimulation test (ACTHST). A low resting cortisol concentration with an inadequate or absent response to synthetic ACTH is consistent with hypoadrenocorticism.^33,34^ The ACTHST protocol was detailed in 34/48 reported cases and varied in timings; however, all were inclusive of resting cortisol and at least one cortisol assessment within 30–180 mins after intravenous (IV) administration of tetracosactide (Table 5). The post-stimulation cortisol level was >60 nmol/l in two cases (60.7 and 63.3 nmol/l in cases 19 and 12, respectively). The current recommended protocol for feline ACTHST is to take a baseline cortisol measurement with two further samples 60 and 90 mins after the ACTH injection.^37^

**Table 5:** ACTH protocols collated from reports of feline hypoadrenocorticism published between 1983 and 2023

Basal/resting cortisol						
30 mins	–	–	–	–	–	–
–	50 mins	–	–	–	–	–
60 mins	–	60 mins	60 mins	60 mins	–	–
–	–	–	120 mins	–	120 mins	120 mins
–	–	–	–	180 mins	–	180 mins
n = 1/34	n = 1/34	n = 15/34	n = 10/34	n = 5/34	n = 1/34	n = 1/34

ACTH = adrenocorticotropic hormone

### Endogenous (plasma) ACTH measurement

Plasma ACTH (endogenous ACTH) was markedly increased in all assessed cases (26/26).^1,4,11,23,24,26,29^ The median plasma ACTH was 2200 pg/ml (range 1223–8000 pg/ml).^1,4,11,23,24^ The RI was in the range of 10–370 pg/ml.^1,4,11,23,24,26^ The Immulite chemiluminescent assay (Siemens) has been validated for endogenous ACTH assessment in cats. The suggested RI while utilising this method is 32–370 pg/ml.^38^

## Treatment

### Acute presentation

The treatment of acute hypoadrenocorticism, as in other species, will depend on the nature of the presentation. Typically, triage, initial testing and stabilisation occur before clinical suspicion of hypoadrenocorticism. Depending on presentation, administration of intravenous fluids (IVFT) will be required, aiming to address hypovolaemia and restore hydration, while decreasing potassium and increasing sodium concentrations. Crystalloids should be prioritised over colloids. In dogs, 0.9% sodium chloride (NaCl) is often recommended; however, recently, the high levels of Na and Cl and the metabolic acidosis associated with 0.9% NaCl have come under scrutiny and balanced crystalloids are more often recommended. The initial treatment for hypovolaemia consists of a 5 ml/kg bolus of crystalloid over 10–15 mins, with reassessment of the cardiovascular status and further bolus if needed. IVFT rates should then be adjusted to effect and as required. Clinical parameters, including heart rate, pulse quality, respiration rate, temperature and systolic blood pressure, and blood parameters, including electrolyte concentrations, creatinine, urea, glucose, albumin and packed cell volume, should be monitored closely within the first 24–48 h, starting at every 2–6 h and spacing out as appropriate, depending on initial results and changes in response to fluids. Acid–base abnormalities are not commonly reported, with metabolic acidosis being described only twice^4,17^ and resolved with IVFT.^30–34^

Hyperkalaemia was reported in the majority of patients (n = 27/47); however, this did not require targeted treatment beyond IVFT in any of the reported cases. If the hyperkalaemia requires specific treatment, then, for cardiac protection, 10% calcium gluconate could be administered slowly (5 mg/kg IV of elemental calcium, which equals approximately 0.5 ml/kg of 10% calcium gluconate). An electrocardiogram should always be utilised during IV administration of calcium to monitor for worsening of bradycardia and shortening of QT intervals, in which case the infusion should be stopped. Such treatment has a relatively quick onset of action (within minutes) and protects the myocardium against cardiotoxicity by altering the threshold potential; however, it is short acting, lasting approximately 20 mins, and does not decrease the serum potassium concentration. Short-acting insulin, in a dose of 0.25–0.5 IU/kg alongside 1 g/kg of diluted 1:4 dextrose followed by 2.5% constant rate infusion (CRI) of dextrose for 6–8 h, can be used to shift potassium intracellularly. Insulin drives potassium into the cells, resulting in a decreased serum potassium level; however, it must be followed by an infusion of dextrose to avoid hypoglycaemia due to the long-acting effect of insulin. The potassium and glucose levels should be monitored regularly. A similar effect can be achieved with sodium bicarbonate in a dose of 1–2 mEq/kg IV (diluted 1:6) administered slowly over 20 mins. Sodium bicarbonate should be used only if blood gas analysis is available in-house, in cases of severe hyperkalaemia and metabolic acidosis with bicarbonate <10 mmol/l. Bicarbonate will alter the pH, increasing binding of calcium to albumin; hence if calcium gluconate was given to the patient initially, its cardioprotective effect can be reduced. Paradoxical cerebral acidosis is a phenomenon that can occur with an increase in blood pH with a concurrent fall in the pH of cerebrospinal fluid during administration of the sodium bicarbonate solution. This is believed to be dose- and rate-dependent, usually transient and clinically irrelevant in patients with adequate ventilation.^39^ Terbutaline and albuterol have been reported to decrease serum potassium concentrations by shifting it intracellularly; however, these medications are not licensed for the treatment of hyperkalaemia.

Hyponatraemia was reported in 32/47 cases. A careful approach should be taken when correcting sodium levels to avoid neurological signs secondary to osmotic demyelination syndrome. Care should be taken when stabilising both sodium and potassium levels, and moving those electrolytes by >0.5 mEq/l/h should be avoided to minimise the risk of osmotic demyelination syndrome and cardiac arrhythmias, respectively.^30^–^34^

Dexamethasone and prednisolone are glucocorticoids commonly used in the treatment of an acute hypoadrenocorticism. They are widely available and absorb rapidly; however, they do not provide mineralocorticoid supplementation. Dexamethasone 0.2–0.4 mg/kg IV is recommended. Hydrocortisone and fludrocortisone have both gluco- and mineralocorticoid potency. Hydrocortisone can be used in a CRI of 0.5 mg/kg/h in an adrenal crisis. An oral formulation is available and a dosage of 0.125 mg/kg q12h can be prescribed under the cascade for maintenance. Hydrocortisone as a glucocorticoid is less potent than prednisolone, with 4 mg of hydrocortisone being equivalent to 1 mg of prednisolone. If desoxycorticosterone pivalate (DOCP) is not available, fludrocortisone can be used and is 125 times more potent a mineralocorticoid than hydrocortisone. The initial recommended dosage is 0.01 mg/kg q12h and no licensed formulations are available in the UK.

### Long-term management

**Figure 2:**
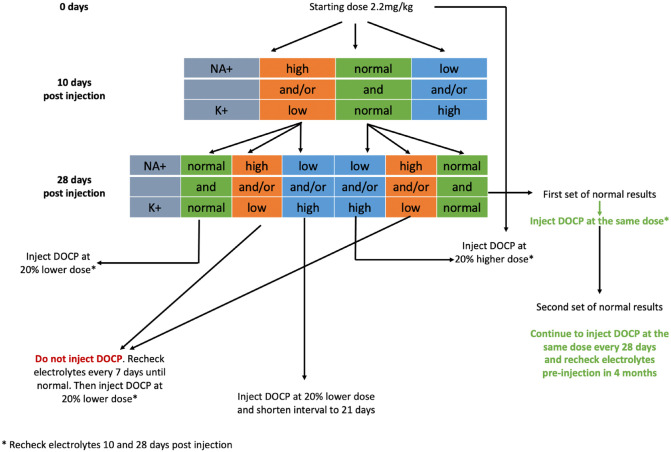
An algorithm for adjusting doses of desoxycorticosterone pivalate (DOCP) in cats with hypoadrenocorticism. Modified from Feldman et al^34^

Long-term treatment, similar to canine hypoadrenocorticism, consists of oral corticosteroids, with several formulations available (prednisolone: 1 mg, 5 mg and 20 mg tablets, liquid formulation of 10 mg/ml oral solution), and mineralocorticosteroids (DOCP), of which variable formulations are available for dogs across the globe; however, their safety has not been assessed in cats.^30–34^ Prednisolone treatment is usually started at 2.5–5 mg/day and recent guidelines for DOCP dosing are available (Figure 2). Great care should be taken when calculating corticosteroid doses and each drug potency must be taken into consideration with regard to the route of administration (Table 6).

**Table 6:** Relative glucocorticoid potencies and their duration of action in cats^40^

	Glucocorticoid potency relative to hydrocortisone	Duration of action (h)
Short acting		
Hydrocortisone	1	<12
Cortisone	0.8	<12
Intermediate acting		
Prednisolone	4	12–36
Methylprednisolone	5	12–36
Triamcinolone	5	12–36
Long acting		
Dexamethasone	30	>48
Bethamethasone	25–40	>48
Paramethasone	10	>48

## Prognosis

The prognosis of feline hypoadrenocorticism is overall reasonable and can be good. A long-term outcome was reported in 39 cats; 7/39 were euthanased. Of these, three failed to respond to treatment within the first 2–5 days of hospitalisation,^4^ and one developed diabetes mellitus, which was challenging for owners to manage.^14^ Three other patients were diagnosed with concurrent conditions as a primary reason for their adrenal insufficiency: two were diagnosed with lymphoma and one with lymphocytic panhypophysitis. It can be concluded that patients with no underlying disease and those with chronic presentation have better outcomes and the long-term prognosis is good. The median survival time cannot be established because of a lack of long-term follow-up; however, the longest reported survival time was 70 months.^4^

Further studies are required to establish the indications for cortisol assessment in feline patients. Retrospective and prospective analyses comparing the efficacy of various ACTHST protocols would be beneficial to achieve a unified diagnostic tool for feline hypoadrenocorticism. Safety and efficacy assessments of DOCP should be considered in future studies.
